# Germline analysis of an international cohort of pediatric diffuse midline glioma patients

**DOI:** 10.1093/neuonc/noaf061

**Published:** 2025-03-12

**Authors:** Marion K Mateos, Pamela Ajuyah, Noemi Fuentes-Bolanos, Sam El-Kamand, Paulette Barahona, Ann-Kristin Altekoester, Chelsea Mayoh, Holly Holliday, Jie Liu, Louise Cui, Elke Pfaff, Alan Mackay, Adam C Resnick, Mark Pinese, Loretta M S Lau, Dong-Anh Khuong-Quang, Kimberly Dias, Catherine Goudie, Alison Salkeld, Jo Lynne Rokita, David T W Jones, Nikoleta Juretic, Elisha Hayden, Stefan M Pfister, Christof M Kramm, Mirjam Blattner-Johnson, Nada Jabado, Maria Tsoli, Orazio Vittorio, Sabine Mueller, Yiran Guo, Katherine Tucker, Sebastian M Waszak, Sebastien Perreault, Chris Jones, Marie Wong-Erasmus, Mark J Cowley, David S Ziegler

**Affiliations:** Children’s Cancer Institute Australia, Lowy Cancer Research Centre, Sydney, New South Wales, Australia; School of Clinical Medicine, UNSW Medicine & Health, UNSW Sydney, Sydney, New South Wales, Australia; Kids Cancer Centre, Sydney Children’s Hospital, Randwick, New South Wales, Australia; Children’s Cancer Institute Australia, Lowy Cancer Research Centre, Sydney, New South Wales, Australia; Children’s Cancer Institute Australia, Lowy Cancer Research Centre, Sydney, New South Wales, Australia; School of Clinical Medicine, UNSW Medicine & Health, UNSW Sydney, Sydney, New South Wales, Australia; Kids Cancer Centre, Sydney Children’s Hospital, Randwick, New South Wales, Australia; Children’s Cancer Institute Australia, Lowy Cancer Research Centre, Sydney, New South Wales, Australia; Children’s Cancer Institute Australia, Lowy Cancer Research Centre, Sydney, New South Wales, Australia; Children’s Cancer Institute Australia, Lowy Cancer Research Centre, Sydney, New South Wales, Australia; Children’s Cancer Institute Australia, Lowy Cancer Research Centre, Sydney, New South Wales, Australia; School of Clinical Medicine, UNSW Medicine & Health, UNSW Sydney, Sydney, New South Wales, Australia; Children’s Cancer Institute Australia, Lowy Cancer Research Centre, Sydney, New South Wales, Australia; School of Clinical Medicine, UNSW Medicine & Health, UNSW Sydney, Sydney, New South Wales, Australia; Children’s Cancer Institute Australia, Lowy Cancer Research Centre, Sydney, New South Wales, Australia; Children’s Cancer Institute Australia, Lowy Cancer Research Centre, Sydney, New South Wales, Australia; Department of Pediatric Oncology, Hematology, Immunology and Pulmonology, Heidelberg University Hospital, Heidelberg, Germany; German Cancer Research Center (DKFZ), Heidelberg, Germany; National Center for Tumor Diseases (NCT) Heidelberg, Heidelberg, a partnership between DKFZ and Heidelberg University Hospital, Germany; Division of Pediatric Glioma Research, Hopp Children’s Cancer Center Heidelberg (KiTZ), Heidelberg, Germany; Division of Molecular Pathology, The Institute of Cancer Research (ICR), Sutton, UK; Children’s Cancer Institute Australia, Lowy Cancer Research Centre, Sydney, New South Wales, Australia; School of Clinical Medicine, UNSW Medicine & Health, UNSW Sydney, Sydney, New South Wales, Australia; Children’s Cancer Institute Australia, Lowy Cancer Research Centre, Sydney, New South Wales, Australia; School of Clinical Medicine, UNSW Medicine & Health, UNSW Sydney, Sydney, New South Wales, Australia; Kids Cancer Centre, Sydney Children’s Hospital, Randwick, New South Wales, Australia; Murdoch Children’s Research Institute, Royal Children’s Hospital, Melbourne, Victoria, Australia; Children’s Cancer Centre, Royal Children’s Hospital, Parkville, Victoria, Australia; Children’s Cancer Institute Australia, Lowy Cancer Research Centre, Sydney, New South Wales, Australia; Division of Hematology-Oncology, Department of Pediatrics, McGill University Health Centre, Montreal, Quebec, Canada; Department of Child Health and Human Development, Research Institute of the McGill University Health Centre, Montreal, Quebec, Canada; Department of Radiation Oncology, Crown Princess Mary Cancer Centre, Westmead Hospital, Westmead, New South Wales, Australia; Sydney West Radiation Oncology Network, Sydney, New South Wales, Australia; Sydney West Translational Cancer Research Centre, Westmead, Australia; School of Medicine, The University of Sydney, Sydney, New South Wales, Australia; Center for Data Driven Discovery in Biomedicine, Children’s Hospital of Philadelphia, Philadelphia, USA; Division of Neurosurgery, Children’s Hospital of Philadelphia, Philadelphia, USA; Department of Bioinformatics and Health Informatics, Children’s Hospital of Philadelphia, Philadelphia, USA; German Cancer Research Center (DKFZ), Heidelberg, Germany; National Center for Tumor Diseases (NCT) Heidelberg, Heidelberg, a partnership between DKFZ and Heidelberg University Hospital, Germany; Division of Pediatric Glioma Research, Hopp Children’s Cancer Center Heidelberg (KiTZ), Heidelberg, Germany; Department of Pediatrics, McGill University, and The Research Institute of the McGill University Health Centre, Montreal, Quebec, Canada; Children’s Cancer Institute Australia, Lowy Cancer Research Centre, Sydney, New South Wales, Australia; Department of Pediatric Oncology, Hematology, Immunology and Pulmonology, Heidelberg University Hospital, Heidelberg, Germany; German Cancer Research Center (DKFZ), Heidelberg, Germany; National Center for Tumor Diseases (NCT) Heidelberg, Heidelberg, a partnership between DKFZ and Heidelberg University Hospital, Germany; Division of Pediatric Hematology and Oncology, University Medical Center Göttingen, Göttingen, Germany; German Cancer Research Center (DKFZ), Heidelberg, Germany; National Center for Tumor Diseases (NCT) Heidelberg, Heidelberg, a partnership between DKFZ and Heidelberg University Hospital, Germany; Division of Pediatric Glioma Research, Hopp Children’s Cancer Center Heidelberg (KiTZ), Heidelberg, Germany; Division of Experimental Medicine, Department of Medicine, McGill University, Montreal, Quebec, Canada; Department of Human Genetics, McGill University, Montreal, Quebec, Canada; Department of Pediatrics, McGill University, and The Research Institute of the McGill University Health Centre, Montreal, Quebec, Canada; Children’s Cancer Institute Australia, Lowy Cancer Research Centre, Sydney, New South Wales, Australia; School of Clinical Medicine, UNSW Medicine & Health, UNSW Sydney, Sydney, New South Wales, Australia; Children’s Cancer Institute Australia, Lowy Cancer Research Centre, Sydney, New South Wales, Australia; Department of Biomedical Sciences, UNSW Medicine & Health, UNSW Sydney, Sydney, New South Wales, Australia; Department of Neurology, Neurosurgery and Pediatrics, University of California San Francisco, San Francisco, California, USA; Department of Pediatrics, University of Zurich, Zurich, Switzerland; Center for Data Driven Discovery in Biomedicine, Children’s Hospital of Philadelphia, Philadelphia, USA; Division of Neurosurgery, Children’s Hospital of Philadelphia, Philadelphia, USA; School of Clinical Medicine, UNSW Medicine & Health, UNSW Sydney, Sydney, New South Wales, Australia; Hereditary Cancer Centre, Nelune Comprehensive Cancer Centre, Prince of Wales Hospital, Randwick, New South Wales, Australia; Swiss Institute for Experimental Cancer Research, School of Life Sciences, École Polytechnique Fédérale de Lausanne (EFPL), Lausanne, Switzerland; Department of Neurology, University of California San Francisco, San Francisco, California, USA; Department of Neurosciences, Centre Hospitalier Universitaire Sainte-Justine, Université de Montreal, Montreal, Quebec, Canada; Division of Molecular Pathology, The Institute of Cancer Research (ICR), Sutton, UK; School of Clinical Medicine, UNSW Medicine & Health, UNSW Sydney, Sydney, New South Wales, Australia; Children’s Cancer Institute Australia, Lowy Cancer Research Centre, Sydney, New South Wales, Australia; School of Clinical Medicine, UNSW Medicine & Health, UNSW Sydney, Sydney, New South Wales, Australia; Children’s Cancer Institute Australia, Lowy Cancer Research Centre, Sydney, New South Wales, Australia; School of Clinical Medicine, UNSW Medicine & Health, UNSW Sydney, Sydney, New South Wales, Australia; Kids Cancer Centre, Sydney Children’s Hospital, Randwick, New South Wales, Australia

**Keywords:** diffuse midline glioma, germline variants, homologous recombination, PARP inhibitor, pediatric

## Abstract

**Background:**

Factors that drive the development of diffuse midline gliomas (DMG) are unknown. Our study aimed to determine the prevalence of pathogenic/likely pathogenic (P/LP) germline variants in pediatric patients with DMG.

**Methods:**

We assembled an international cohort of 252 pediatric patients with DMG, including diffuse intrinsic pontine glioma (*n* = 153), with germline whole genome or whole exome sequencing.

**Results:**

We identified P/LP germline variants in cancer predisposition genes in 7.5% (19/252) of patients. Tumor profiles differed, with the absence of somatic drivers in the PI3K/mTOR pathway in patients with germline P/LP variants versus those without (*P = *.023). P/LP germline variants were recurrent in homologous recombination (*n* = 9; *BRCA1, BRCA2, PALB2*) and Fanconi anemia genes (*n* = 4). Somatic findings established that the germline variants definitively contributed to tumorigenesis in at least 1% of cases. One patient with recurrent DMG and pathogenic germline variants (*BRCA2*, *FANCE*) showed a near-complete radiological response to PARP and immune checkpoint inhibition.

**Conclusions:**

Our study determined the prevalence of pathogenic germline variants in pediatric DMG and suggests a role in tumorigenesis for a subset of patients.

**Key Points**

- In total, 7.5% of DMG patients carried pathogenic germline variants in cancer predisposition genes.
- DMG genomes supported a tumorigenic role in 1% of cases.
- A patient with *BRCA2/FANCE*-associated DMG showed a near-complete response to PARP and immune checkpoint inhibitors.

**Importance of the Study**

This is the largest international study of germline variants in DMG conducted to date. Germline pathogenic/likely pathogenic (P/LP) variants in cancer predisposition genes occurred in 7.5% (19/252) of patients with DMG and were enriched in DNA damage repair genes. DMG genomes provided support for the contribution of germline variants to tumor development in 1% of the cohort. However, further somatic associations were identified across the cohort. Molecular tumor profiles of patients with and without a P/LP germline variant differed, with a lack of somatic PI3K/mTOR pathway alterations in patients with P/LP germline variants. One patient with germline *FANCE* and *BRCA2* pathogenic variants had a near-complete radiologic response after treatment with PARP inhibitors and an immune checkpoint inhibitor. Further work is needed to understand how pathogenic germline variants and somatic driver alterations contribute to the development of DMG and therapeutic vulnerability.

Diffuse midline gliomas (DMG) are aggressive high-grade glial childhood cancers with a dismal prognosis.^1,2^ Diffuse intrinsic pontine gliomas (DIPG) are a subset of DMG tumors located in the pons. Treatment options for DMG are essentially palliative, meaning that most children die from their disease 9–12 months from diagnosis.^1,3^ The prevalence of pathogenic/Likely pathogenic (LP; P/LP) germline variants in cancer predisposition genes (CPGs) in DMG is unknown and may offer new insights into oncogenesis and patient management.

Improved understanding of germline determinants of childhood cancer has helped in the management and treatment of high-risk pediatric cancers.^4,5^ Landscape studies in pediatric malignancies have detected germline P/LP variants in 5%–16% of children,^5–7^ and reaching as high as 40% in specific cancers such as SHH-activated medulloblastoma.^8^ Understanding these associations provides insights into tumorigenesis and has implications for clinical germline testing for patients and families. Germline findings can have important therapeutic implications, such as the discovery that children with gliomas with underlying constitutional mismatch repair deficiency have improved survival using immune checkpoint inhibitors.^4,9^

Since 2016, there have been case reports of children with midline high-grade gliomas reported to carry germline P/LP variants in CPGs.^10,11^ Two studies detected germline P/LP variants in *MUTYH*^11,12^ in children treated for DIPG/DMG, while another described germline findings in *ATM, FANCM,* and *MYCN* in 3 children with DMG.^13^ A preprint of exome germline analysis for the first patients on the BIOMEDE trial reported germline P/LP variants in *ATM, SEC16A, SELPLG, KMT2D,* and other loci.^14^ These reports suggest that children with DMG may have a genetic tumor syndrome, highlighting the need for a multicentre study of the prevalence of germline P/LP variants in DMG.

Here, we sought to investigate the prevalence of germline P/LP variants in CPGs in an international cohort of patients consecutively diagnosed with biopsy-proven DMG or DIPG. The secondary aims were to compare clinical and survival outcomes; and somatic mutational and transcriptional signatures for patients with and without underlying germline findings. We also sought to determine whether germline P/LP variants can have therapeutic implications for DMG patients through preclinical modeling and review of individual treatment strategies.

## Methods

### Patients and Samples

This international multi-institutional study had a total of 252 patients including cohorts from the Australian Zero Childhood Cancer (ZERO) Precision Medicine Program (*n* = 46), Children’s Brain Tumor Network (CBTN) including the Pediatric Pacific Neuro-oncology Consortium (PNOC; *n* = 52), German Cancer Research Center (DKFZ; *n* = 66), The Institute of Cancer Research (ICR; HERBY, BIOMEDE, Taylor; *n* = 74) and The Research Institute of the McGill University Health Centre, Montreal (*n* = 14). Patients were consecutively diagnosed with the radiological diagnosis of DIPG (and were either H3 K27-altered or wild type) or with histologically confirmed DMG (H3 K27-altered tumors occurring in midline structures outside the pons), up until December 31, 2020. Patients who did not have germline sequencing data available were excluded. Each center submitted de-identified data with clinical information, including diagnostic, treatment, survival data, and paired somatic and germline molecular data. All centers have approved data access and informed consent was obtained from all patients or legal guardians.

### Sequencing

Each group performed germline DNA sequencing with Illumina technology including ZERO (46 samples by whole genome sequencing [WGS]), CBTN (36 WGS and 16 whole exome sequencing [WES]), DKFZ (66 WES), ICR (HERBY (25 WES), BIOMEDE (29 WES), Taylor [20 WGS]), and Montreal (5 WGS, 9 WES). Detailed methodology for these studies has been previously reported.^3,7,15–20^

Germline analysis average minimum sequencing depth varied according to the WGS or WES platform used and is listed with somatic sequencing depth in Table 1. Whole transcriptome RNA sequencing (RNA-seq) was performed on the samples acquired from ZERO using a TruSeq stranded mRNA preparation kit and sequenced on either the Illumina HiSeq 2000 to 40 M reads (*N* = 10) or the NextSeq 500 to 80 M reads (*N* = 29). Detailed RNA-seq methodology has been previously reported.^7^

**Table 1.** Sequencing Depth and Sample Numbers Across the 5 International Cohorts

Cohort	*N* samples	Somatic sequencing	Avg min sequencing depth (x)	Germline sequencing	Avg min sequencing depth (x)	Germline WGS	Germline WES
ZERO	46	WGS	90	WGS	30	46	0
DKFZ	66	WES	150	WES	100	0	66
Montreal	14	WGS/WES	90	WGS/WES	30	5	9
CBTN	52	WGS	60	WGS/WES	30	36	16
ICR	54	WES	90	WES	30	0	54
ICR	20	WGS	30	WGS	30	20	0
**TOTAL**	**252**					**107**	**145**

Legend: “N,” number; “WES,” whole exome sequencing; “WGS,” whole genome sequencing. “Avg min sequencing depth,” the average minimum sequencing depth. Bold text indicates total number for samples, germline WGS and germline WES.

DNA methylation analysis was conducted using the Illumina Infinium MethylationEPIC 850K microarray. Raw data were analyzed in R (v4.2.2; https://www.R-project.org/) using the Bioconductor packages limma (v3.52.4)^21^ and minfi (v1.42.0),^22^ which assessed for quality with poor performing probes removed, batch corrected, and normalization performed (Supplementary Methods).

### Pipeline

All raw sequencing data were run through either the ZERO or DKFZ bioinformatic pipelines for their local data, which have been extensively described previously.^7,19^ Variant call format (VCF) files and variants called by the DKFZ algorithm were then run through the internal ZERO bioinformatic pipeline for harmonization of variant analysis and curation.

### Gene Selection

A manually curated cancer predisposition gene of interest list of 191 genes was used in germline variant analysis (Supplementary Table 1).

Genes were annotated with a traffic light assignment based on clinical relevance. A traffic light system, developed by variant curators for germline genes, defines a gene of interest and assigns the clinical relevance as follows: green traffic light for highly relevant CPGs, amber for those with intermediate clinical relevance in childhood cancer and red were genes were excluded from the analysis (lack of clinical evidence or contraindicatory evidence at the time of manual gene curation, January 2022).

Finally, the variants were filtered and ranked according to the helium score. Helium, an in-house software scoring system that follows recommendations from ACMG guidelines, generated a “helium score” that was combined with other factors to predict which variants were likely benign versus pathogenic. Helium scored the germline variants by those that were rare in the annotated controls (<1% frequency in the population), were previously annotated as LP or pathogenic in ClinVar or were novel loss-of-function variants in tumor suppressor genes (Supplementary Figure 7). *In silico* prediction tools, including Polyphen and SIFT annotated by VEP v100, and PROVEAN, FATHMM, MetaSVM, and MetaLR annotated by dbNSFP(v4.0a), were used to support the pathogenicity assessment of missense variants using a consensus approach of at least 4 of the 6 in silico predictors.

### Variant Filtration

The VCF files analyzed included single nucleotide variants and small insertion-deletion variants (indels). Copy number variant analysis was also performed for the Australian ZERO data. To aid with the prioritization of germline variants for curation, only variants with a green traffic light and a helium score over 99 (excluding splice variants) were curated. These were the variants that were predicted as pathogenic/LP or were novel loss-of-function variants in tumor suppressor genes, with support from in silico prediction tools described above. Additionally, for recessive tumor genes, variants needed a VAF above 0.6 or to be compound heterozygous to be manually curated. However, 3 exceptions for autosomal recessive genes were analyzed with only a single hit in the germline for *MUTYH, CHEK2,* and Fanconi anemia genes due to potential dosage sensitivity.

### Variant Curation

Only variants in coding regions or splice region variants were examined in this study. Variant curation was performed manually according to the ACMG/AMG 2015 guidelines by molecular scientists.^23^ Variants were classified as Pathogenic (P), LP, Variant of unknown significance (VUS), Likely benign or Benign (B). For the purposes of this paper, we refer to germline pathogenic/LP variants as “germline P/LP variants” or “deleterious variants” (loss or gain of function). Germline P/LP variants in CPGs were interpreted based on the available clinical and tumor molecular features. Relevant germline findings were then manually curated by a second reviewer.

We included “VUS with hypothetical function effect as per functional grading (score 3.8)” in the list of variants for manual curation.^24^ These variants are predicted to have a potential functional impact, more than a standard “VUS,” therefore leaning towards the spectrum of an LP variant. These were manually reviewed on a case-by-case basis following the application of the clinical grading score. Variants that were reported in this study were variants that were P, LP, or VUS with functional score ≥3 and clinical score ≥1.

### Mutational Signatures

Mutational signatures were assessed for correlation with the presence of deleterious germline variants in the corresponding pathway. Analysis was performed using COSMIC v2 and COSMIC v3 signatures.^25^ For example, mutational signature 3 (Sig3 in COSMIC v2, SBS3 in COSMIC v3) was evaluated in association with the presence of deleterious variants in the HR pathway. The contribution of COSMIC v3 signatures to the mutational profile of each whole-genome sequenced sample was evaluated using Sigminer (v2.2.2).^26^ Single base substitution, indel, and copy number signatures were all examined; however, doublet signatures were excluded due to the low mutational burden of most samples.

### Genome-Wide Loss of Heterozygosity

Genome-wide loss of heterozygosity (LOH) was quantified as the proportion of the autosomal genome where the minor allele copy number fell below 0.5. Proportions were calculated using a total autosomal genome size of 2 881 033 286 bp. We evaluated the association between somatic mutations in *TP53* and genome-wide LOH proportion using a single-tailed Wilcoxon rank sum test. The adjusted *P* values reported were corrected for multiple tests using Holm’s correction method.

### Genotype–Phenotype Statistical Analysis

Progression-free and overall survival were assessed using the Kaplan–Meier method (log-rank test, significance level *P* < .05). Additional genotype–phenotype correlations were examined for key variables including age at diagnosis, sex, tumor location, and presence of *TP53* somatic mutation. Categorical variables were compared using the chi-squared or Fisher’s exact test; continuous variables were compared using logistic regression. Two-tailed *P* values <.05 were significant. Statistical analysis was conducted in R, Version 2022.12.0 + 353.

### Cell Culture

SU-DIPGXIII cells were maintained in Neurocult NS-A proliferation media (StemCell Technologies) supplemented with 1X Antibiotic-Antimycotic (ThermoFisher), hFGF (10 ng/mL), hEGF (20 ng/mL), and heparin (0.0002%; StemCell Technologies) on flasks coated with poly-L-ornithine (0.01%; Sigma) and laminin (10µg/mL; Sigma). HEK293T cells were maintained in DMEM (ThermoFisher) supplemented with 10% FBS (ThermoFisher) and GlutaMAX (ThermoFisher). All cell lines were cultured at 37 °C in a 5% carbon dioxide incubator. Cells tested negatively for mycoplasma contamination and were confirmed to match the original tumor by STR profiling. SU-DIPGXIII cells (H3.3 K27M) were *TP53* mutant and *MYCN* amplified,^27^ obtained from Nada Jabado (Canada) and authenticated at the Garvan Molecular Genetics Core Facility.

### Generation of Inducible BRCA1 Knockdown (KD) Cells

TRIPZ Inducible Lentiviral plasmids containing shRNA targeting *BRCA1* were purchased from Dharmacon (shRNA #1 TATGTGGTCACACTTTGTG, shRNA #2 TTCAGTACAATTAGGTGGG). GIPZ Non-silencing Lentiviral shRNA (Dharmacon) was used as a negative control. HEK293T cells were co-transfected with lentiviral constructs and Trans-Lentiviral packaging mix (Dharmacon) using Lipofectamine 3000 reagents (ThermoFisher) according to the manufacturer’s protocol (Supplementary methods).

To induce *BRCA1* knockdown (KD), cells were treated with 1 µg/mL doxycycline. Knockdown of *BRCA1* was verified using western blotting (Supplementary methods).

### Treatment Regimens

SU-DIPGXIII cells were seeded adherently in triplicate wells (1200 cells per well) in 6-well plates coated with poly-l-ornithine (0.01%) and laminin (10 µg/mL) in media with or without 1 µg/mL doxycycline to induce *BRCA1* KD. After 72 hours, cells were treated with 1 µg/mL niraparib (Selleck Chemicals). Cells were then incubated for 1 week with niraparib replenished in the media after 3 days. The resulting colonies were stained with MTT (Sigma; 1 mg/mL) and imaged on a BioRad GelDock. Colonies in each well were counted using ImageJ software, normalized to the average of the untreated control wells and technical triplicates were averaged. Three independent experiments were performed. An ANOVA test with Tukey’s or Sidak’s multiple comparisons test was used to test for statistical significance.

## Results

### Clinical Characteristics of the International DMG Cohort

We collected data from 252 DMG patients including germline and somatic sequencing data, demographic and clinical information (Table 2). The cohort included 153 patients with DIPG (defined by imaging criteria) and 99 patients with non-pontine DMG, H3 K27-altered. The median age of the international cohort was 8 years (range 0.7–46 years), with an even distribution between males (111/220; 50.5%) and females (109/220; 49.5%; data not available for 32 patients). The median overall and progression-free survival for the cohort was 11 and 6.5 months, respectively. Tumor location was predominantly in the pons (153/219; 69.9%), with the next most common location in the thalamus (47/219; 21.5%) followed by spinal tumors (6/219; 2.7%). Most tumors were H3 K27-altered (204/216; 94.4%), and a minority H3 K27 wild type (12/216; 5.6%, [all DIPG patients]; Table 2). H3 K27 status was not available for 36 patients with DIPG.

**Table 2.** Baseline Characteristics of DMG Patient Cohort

Patient characteristics (*n* = 252)			
Median age at diagnosis	8 (0.7–46)		
(years, range)			

Variable	Categories	*N* affected/total	%
Sex	Male	111/220	50.5
	Female	109/220	49.5
Diagnosis	H3 K27 Altered	204/216	94.4
	H3 K27 Wild type	12/216	5.6
Location	Pons/Brainstem	153/219	69.9
	Thalamus	47/219	21.5
	Spine	6/219	2.7
	Other*	13/219	5.9
Somatic TP53 variant	Yes	118/188	62.8
	No	70/188	37.2
Germline P/LP variant	Yes	19	7.5
	No	233	92.5
Median overall survival	11 (0–100)		
(months, range)			
Median progression-free survival	6.5 (0–90)		
(months, range)			
Status at last follow-up	Alive/censored	29/168	17.3
	Deceased	139/168	82.7

Treatment characteristics		*N* affected/total	%
Upfront treatment	Radiation	194/203	46.3
Adjuvant treatment	Chemo/targeted	119/203	58.6

*N*, Number of patients; Total, total of cases with respective data available. Other*: concurrent brainstem and spinal tumor (*n* = 1), concurrent brainstem + suprasellar (*n* = 1), suprasellar (*n* = 1), pineal (*n* = 1), basal ganglia (*n* = 1), cortical (*n* = 2), infratentorial locations including cerebellum that do not occur with contiguous thalamic or brainstem lesion (*n* = 4), other location not otherwise specified (*n* = 1). P/LP variant, pathogenic/likely pathogenic variant.

Where detailed treatment data was available (*n* = 203), 194 patients received upfront radiotherapy, 7 patients did not receive radiotherapy at any stage, and 2 were unknown. Adjuvant chemotherapy/targeted therapy was administered for 119 patients, with 29 not receiving adjuvant therapy (data not available in 55). One hundred and fifty-eight patients had both H3K27 status and survival data available (153, H3 K27-altered; 5 H3 K27 wild type). The difference in overall survival was statistically significant (*P* = .022) for H3 K27-altered vs wild-type status (Supplementary Figure 1).

### Prevalence of Deleterious Germline Variants in Patients With DMG

Germline whole genomes–exomes were evaluated using our bioinformatic pipeline that incorporated a curated list of CPGs. Following this, manual curation of germline P/LP SNV and small indels was performed. We identified 19 patients with 21 germline P/LP variants in CPGs (7.5%; Table 2, Supplementary Figure 2). There was one variant of uncertain significance (VUS) with a functional score of 3 in *BRIP1* (Supplementary Methods). This VUS subtype (functional score of 3) is classified by a hypothetical functional effect based on either molecular predictions or due to de novo occurrence and provides supporting evidence of pathogenicity if there is genotype–phenotype correlation.^24^ The prevalence of germline findings was 10/150 (6.7%) in patients with H3.3 mutant tumors and 3/32 (9.4%) in patients with H3.1 mutations, which was not significantly different (*P* = .70).

The prevalence of P/LP germline variants was not significantly different according to location, comparing pontine tumors versus non-pontine tumors (prevalence of P/LP germline variants for tumors located in the pons 10/153 (6.5%), thalamus 5/47 (10.6%), spine 0/6, or other 1/13 (7.7%); *P* = .74). The presence of H3K27 alteration compared to wild type was not significantly associated with presence of germline P/LP variants (H3 K27*-*altered, 16/204 (7.8%); wild type, 0/16 (0%), *P* = .61). There was no association with age nor sex in relation to the presence of a germline P/LP variant in CPGs (*P* = .06, *P* = .82, respectively). There was no difference in progression-free and overall survival for patients with and without germline P/LP variants (Supplementary Figure 3). For those patients with somatic *TP53* sequencing results, 62.8% (118/188) harbored a somatic pathogenic *TP53* variant, but there was no association between the presence of a somatic *TP53* variant and a pathogenic germline variant (*P* = .21). No patients in the cohort had a germline P/LP *TP53* variant.

### Enrichment of Germline Deleterious Variants in the DNA Damage Signaling Pathways

The largest group of germline findings was in genes involved in the homologous recombination pathway (HR; 9/21 = 43.9%). This included *PALB2* (*n* = 3), *BRCA2* (*n* = 2), *CHEK2* (*n* = 2), *BRCA1* (*n* = 1) and *BRIP1* (*n* = 1; Supplementary Figure 1). Other altered DNA repair mechanisms included the Fanconi anemia pathway (*FANCE, FANCD2, FANCM, SLX4;* 4/21 = 19%) and mismatch repair pathways (*PMS2, MSH2;* 2/21 = 9.5%). Other P/LP germline variants were distributed across genes involved in a variety of different pathways: MAPK-ERK signaling (*LZTR1* [*n* = 2]), receptor tyrosine kinase signaling (*FGFR2*), metabolism (*SDHA*), epigenetics (*MAX*), and transcriptional regulation (*HOXB13*).

### Integration of Tumor-Germline Analysis

Next, we evaluated whether the germline findings correlated with somatic variants and genome-wide mutational signatures in tumors of patients with or without germline P/LP variants.

**Figure 1.**
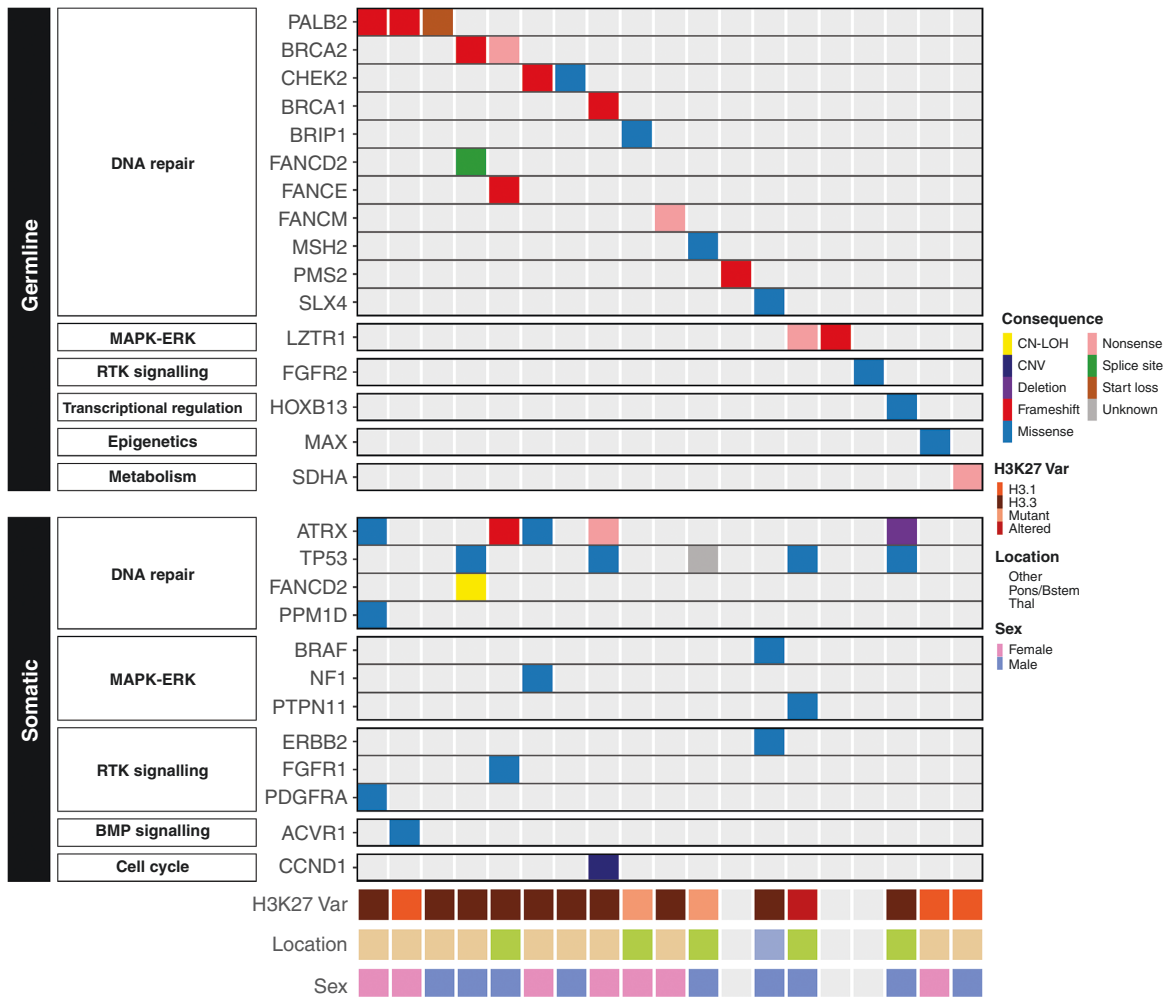
Germline and somatic landscape of diffuse midline gliomas (DMG) patients with germline deleterious variants. Accompanying somatic pathogenic variants are depicted for 19 patients with germline P/LP variants and DMG. While somatic HR defects were present, only one patient had a second somatic hit in an HR locus (*FANCD2*) also seen in the germline. There was an absence of PI3K-AKT-mTOR somatic deleterious variants. “HR,” homologous recombination; “MMR,” mismatch repair; “BER,” base excision repair; “MAPK-ERK,” “mitogen-activated protein kinase - extracellular-regulated kinase”; “RTK,” receptor tyrosine signaling. “PI3K-AKT-mTOR,” “phosphatidylinositol-3-kinase - Ak strain transforming- mammalian target of rapamycin”; “BMP,” “bone morphogenetic protein.” “H3K27 var,” H3K27 variant; “Mutant” refers to patients with a known somatic H3K27M mutation who do not have further details regarding the type of histone mutation; “Altered” refers to H3K27 altered without H3K27M mutation

We first sought to identify somatic variants present in patients with and without P/LP germline variants who had DMG whole genome, whole exome, or panel sequencing data (*n* = 200). Only one patient had a somatic hit in the same locus as the germline pathogenic variant (*FANCD2*) due to CN-LOH. We therefore next evaluated the other somatic drivers in our DMG cohort. Somatic drivers in DMGs with germline P/LP variants were identified in 9/14 (64%) cases, with the somatic changes affecting DNA damage signaling, epigenetics, MAPK-ERK, and receptor tyrosine kinase signaling (Figure 1). The most common somatic pathway altered was in DNA damage signaling, including variants in *ATRX* and *PPM1D* (accounting for 6/17 of the total somatic variants and detected in 4/14 patients with available data). The second most common pathway altered in these tumors was MAPK-ERK signaling (*BRAF, NF1, PTPN11;* 3/17 variants in 3 patients). Of the 186 patients without germline P/LP variants and available cancer genomes–exomes, 41/186 patients had a somatic variant in the DNA damage response pathway (*ATRX, ATM, PPM1D, POLE*) and 21/186 had a variant in the MAPK-ERK pathway (*BRAF, NF1, PTPN11, MAPK12*). There was no statistically significant difference between the germline-affected and unaffected patients with respect to variants affecting the DNA damage response or MAPK-ERK pathways (*P *= .52*, P *= .38, respectively). However, notably, somatic drivers in the PI3K-AKT-mTOR pathway were significantly less common in patients with a germline P/LP variant (0/19, 0%), compared with other patients (50/186, 26.9%; *P* = .023). *ACVR1*, a commonly altered gene in H3.1 mutant DMG^16^ was identified in the tumor of only 1 patient (H3.1 K27M) with a germline *PALB2* deleterious variant compared to 27/186 (14.5%) patients who had somatic *ACVR1* pathogenic variants detected in the non-germline mutant group, although this was not statistically significant (*P* = .70). Thus while only one patient had a second somatic hit in the same gene as the germline pathogenic variant, the somatic landscape of patients with germline variants differed from other patients.

**Figure 2.**
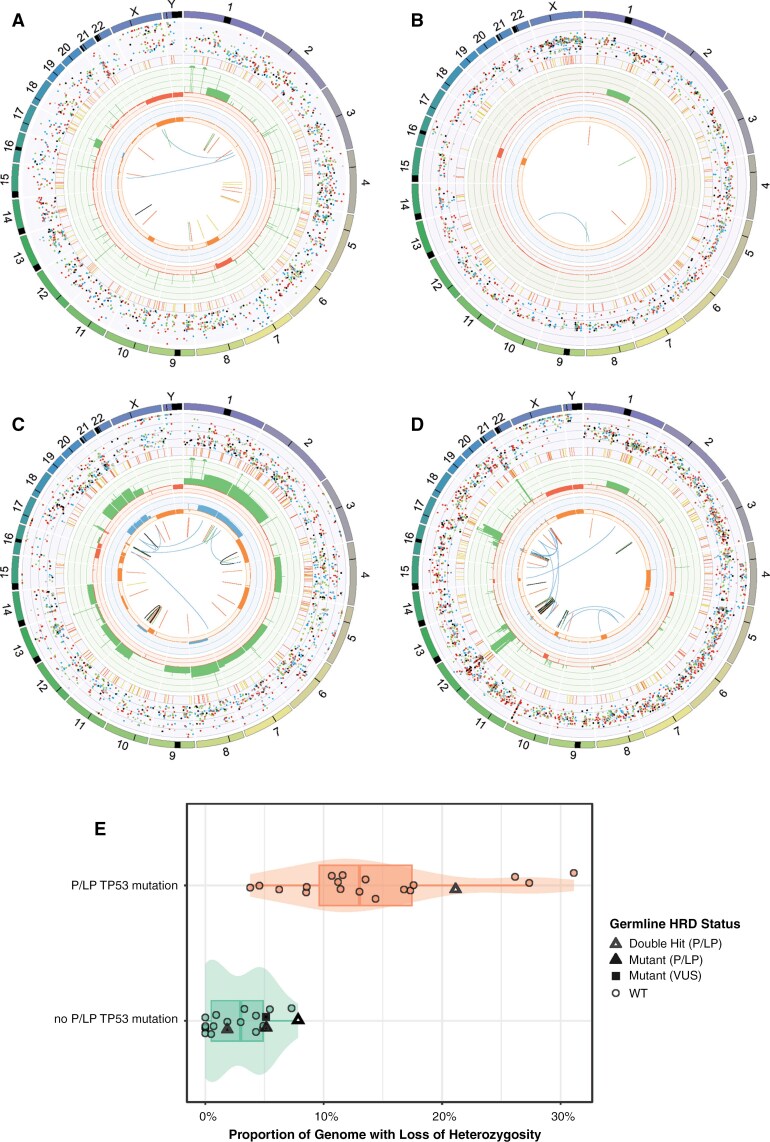
The germline landscape contributes to somatic copy number aberrations.. Four patients with deleterious germline variants (*PALB2* [*n = 2*]), *BRCA2* and *FANCE* [*n = 1*]*, BRCA2* and *FANCD2* [*n = *1]) and corresponding circos plots of somatic variants are depicted (A–D), including copy number gains (green) and copy number losses (red). There are varying levels of genome instability, with more copy number changes observed in Patients C and D who have 2 deleterious germline variants each. (E) Contribution of somatic *TP53* pathogenic/likely pathogenic (P/LP) variants to genome-wide loss of heterozygosity (LOH). Patients with somatic *TP53* P/LP variants had a significantly increased genome-wide LOH compared to somatic *TP53* wild-type patients (*P *< .0001). Patients who have an underlying germline P/LP variant in an HR-related gene (termed homologous recombinant deficiency (HRD) in this figure) are depicted as triangles, and colored white for patients harboring multiple P/LP germline HRD mutations. In *TP53* wild-type patients, while HRD mutants had higher median genome-wide LOH proportions than HRD intact patients, this trend was not significant at α = 5% (*P* = .059). “HRD,” homologous recombination deficiency, refers to those patients with elevated mutational signature 3 based on COSMIC v2. “VUS,” variant of unknown significance in *BRIP1*.

We next examined the somatic mutation profiles of 46 patients with DMG for whom raw DNA sequencing data (WGS or WES or targeted panel sequencing) was available. There were no notable differences between the genomic tumor profiles of the 4 patients with deleterious germline variants compared to 42 patients without deleterious germline variants (Figure 2A–D). However, 2 patients with both *BRCA2* and either *FANCD2* (Figure 2C) or *FANCE* (Figure 2D) germline deleterious variants also had complex genomic tumor profiles with numerous copy number gains, losses, and copy-neutral LOH (CN-LOH) events. Both tumors had other somatic drivers (*TP53* and *ATRX,* respectively) that may have contributed to the different phenotypes (Figure 2C–D).

RNA sequencing was available for the 4 patients with germline P/LP variants, thus we next assessed the impact on gene expression of these variants. The patient with a germline *FANCD2* variant (Figure 2C) had a second hit in the tumor through chromosome 3 LOH, as described above. This was confirmed in the tumor RNA with homozygous alternate splicing of *FANCD2* as a result of the mutation. Low expression of *BRCA2* was also observed in the tumor suggesting loss of BRCA2 function. The patient with germline *BRCA2* and *FANCE* pathogenic variants had a loss of expression of both *BRCA2* and *FANCE* in the tumor, suggesting loss of BRCA2 and FANCE function. For the 2 patients with germline variants in *PALB2*, one showed expression of their heterozygous *PALB2* variant in the tumor RNA, indicating that there was no second hit in the tumor. Whereas the other patient with a pathogenic *PALB2* variant, RNA-seq revealed the mutant allele was not transcribed, resulting in gene expression of only the wild-type allele. Therefore, 2/4 patients with germline P/LP variants, with WGS and whole transcriptome data, had a second hit seen in the tumor as evidenced by CN-LOH or complete loss of gene expression.

### Integration of Tumor-Germline Analysis: Chromosomal Instability

Given the complex genomic changes seen in the 2 patients with 2 pathogenic germline variants each (Figure 2C, D), we sought to determine if the germline variants contributed to this somatic genotype, or whether somatic *TP53* pathogenic variants alone were a driver of this tumor profile.^28^ We used genome-wide LOH as a marker of genomic instability.^29^ Genome-wide LOH can occur as a result of copy-loss LOH or copy-neutral LOH with duplication of the remaining allele. We confirmed that somatic *TP53* P/LP variants were associated with an increased median percentage of genome-wide LOH compared to intact *TP53* patients (*P *< .0001; Figure 2E). Tumors with germline deleterious variants in HR-related genes also had a higher median proportion of genome-wide LOH, although this trend did not reach statistical significance (*P* = .059). Regardless of the *TP53* status, the presence of 2 germline P/LP variants resulted in the highest proportion of genome-wide LOH within those that had a germline deleterious variant (Figure 2E). This suggests that germline deleterious variants in HR-related genes affect the somatic mutation landscape and contribute to genome-wide LOH, especially when 2 separate heterozygous P/LP germline variants are present in different genes in the same patient.

### Mutational Signatures

To further evaluate the impact of germline variants on the tumor genome, we next investigated mutational signatures.^30^ We first sought to determine if there was an association between germline variants in the HR pathway and the presence of mutational signature 3 (“signature 3” in the COSMIC v2 signature catalog).^31^ Signature 3 is associated with HR deficiency in breast and ovarian cancer^32,33^ and medulloblastoma,^5^ but its relevance in pediatric glioma remains unclear. Of the 40 cases with WGS and derived mutational signatures, the 3 cases with the highest levels of signature 3 (accounting for 33.8%–38.8% of the tumor’s somatic variants) included the 2 cases with a pathogenic germline *BRCA2* variant (Supplementary Figure 4). The third case with high signature 3 levels, did not have a clear explanation for this signature including no detectable germline or somatic P/LP variants in HR genes. RNA-seq analysis of this third case demonstrated low expression of *BRCA1* in the tumor (patient *BRCA1* expression: 1.76 transcripts per million (TPM) compared with the cohort median TPM 6.3 [range 1.13–16.94]). We therefore investigated the DNA methylation profiles of the *BRCA1* and *BRCA2* promoters. There was no discernible difference in the DNA methylation status of CpG islands at the promoter or body of *BRCA1* or *BRCA2* for the 3 cases with high signature 3 compared to the rest of the cohort (Supplementary Figure 5).

Using a recently updated and refined signature catalog (COSMIC v3),^25^ we found that of the 4 patients with germline P/LP variants and signature data, only those harboring deleterious mutations in 2 HR genes possessed signatures associated with HR deficiency. The patient with germline *BRCA2* and *FANCE* variants demonstrated elevated signature 3 (known as SBS3 in this catalog), and the patient with germline *BRCA2/FANCD2* variants showed elevated contributions of copy number signature 17, although this signature might also be affected by this patient’s somatic *TP53* mutation. No patients with germline P/LP variants in HR-related genes showed elevated ID6; however, those harboring multiple germline P/LP variants (*BRCA2/FANCE, BRCA2/FANCD2)* had above average contributions of indel signature 8, which may indicate increased repair by non-homologous activity pathways that are preferred when HR pathways are compromised. Together these data suggest that germline variants in HR genes may contribute to tumorigenesis in a subset of DMG cases. To sum up, 2 patients had evidence of a second somatic hit illustrated by CN-LOH or loss of RNA expression, demonstrating cooperation between germline and somatic alterations. Therefore 2/200 patients (1%) where somatic data was available had evidence of a definitive impact of the germline variant on tumorigenesis, with the impact in other patients remaining undefined.

### Treatment Implications for Patients With Pathogenic Germline Variants

Given the prevalence of HR defects in the germline and somatic analysis, we next sought to determine potential therapeutic relevance. To model deleterious variants in HR-related genes in DMG, we generated SU-DIPGXIII (H3.3K27M) cells with inducible *BRCA1* knockdown (KD; Supplementary Figure 6A). We found that DMG cells with knockdown of *BRCA1* were more sensitive to the PARP inhibition when compared to uninduced cells with intact *BRCA1* expression (Supplementary Figure 6B–C; *P* = .005, *P* = .0067 for shRNA constructs #1 and #2, respectively).

**Figure 3.**
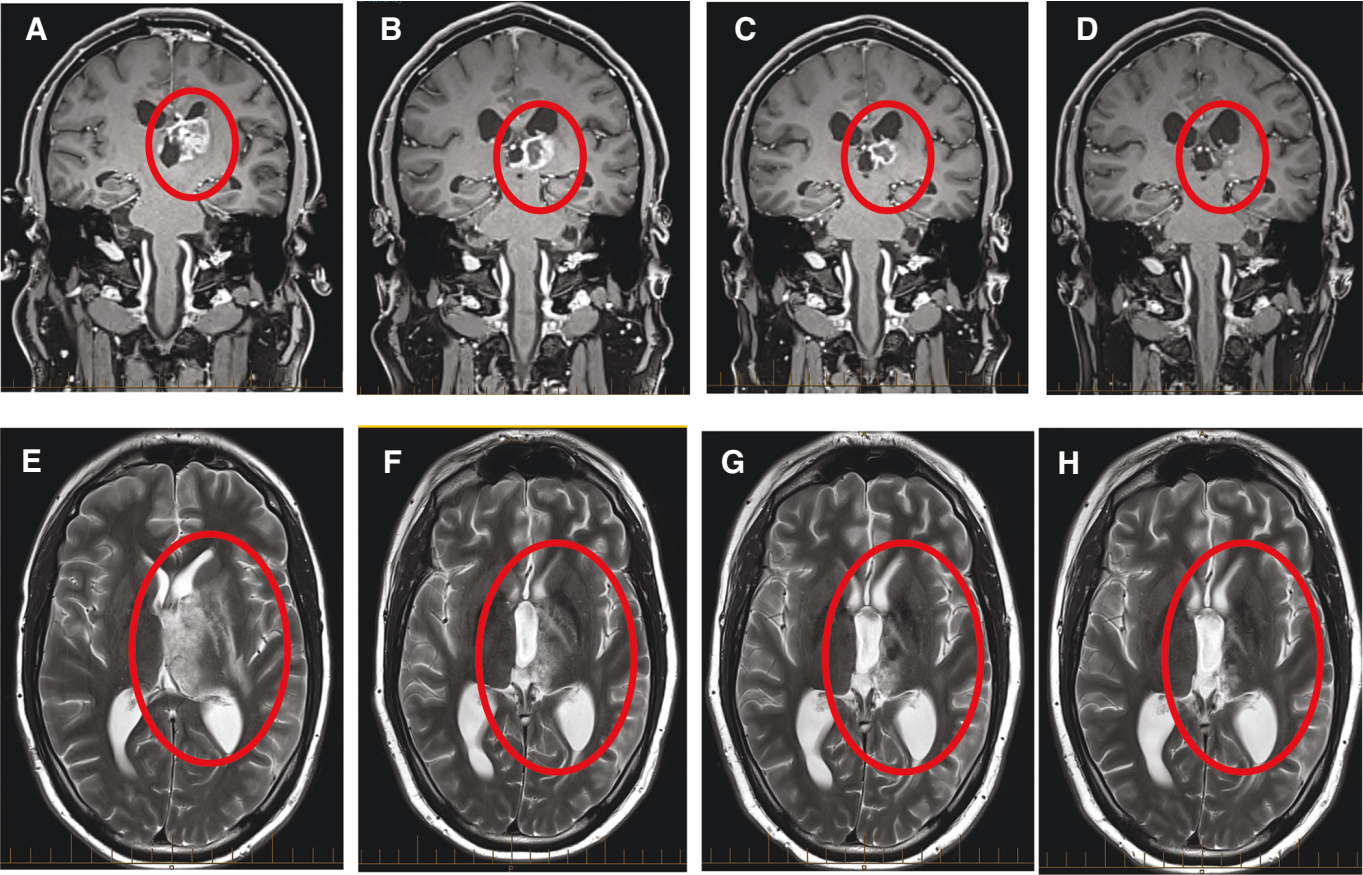
Radiologic response to PARP inhibition in a diffuse midline glioma (DMG) patient with pathogenic germline variants in *BRCA2* and *FANCE.* T1-weighted MRI brain with contrast in a patient with relapsed DMG, germline *BRCA2,* and *FANCE* pathogenic variants, following treatment with a PARP inhibitor and durvalumab at (A) pretreatment, (B) 6 weeks, (C) 2.5 months and (D) 5 months after commencement of therapy. Axial non-contrast T2-weighted images show the same time points in panels E, F, G, and H, respectively.

We next identified one patient who had received treatment targeted towards the germline findings. A young adult with a thalamic H3.3 K27M mutant DMG was treated with surgical resection and focal irradiation to 53.4 Gy. Three months after diagnosis, local recurrence led to a second resection. Disease recurrence was confirmed via biopsy. This patient did not receive re-irradiation. Sequencing revealed pathogenic germline *BRCA2* and *FANCE* variants and high signature 3 (38%, COSMIC v2). A second recurrence was confirmed 8 weeks following the re-resection (Figure 3A). The patient was enrolled in a clinical trial combining the PARP inhibitor olaparib with the PD-L1 inhibitor durvalumab due to the germline and somatic findings (Study ID: ACTRN12617001000392).^34^ The patient had a partial response by RANO 6 weeks after starting treatment (Figure 3B), with clinical and radiological improvement after 2.5 months (Figure 3C) and near-complete tumor response after 5 months of therapy (Figure 3D), suggesting the potential impact of germline HR variants on clinical response to PARP inhibition in DMG patients. Unfortunately, this patient relapsed 8 months after initiation of salvage therapy.

### Screening Implications: Family History of Cancer

In the ZERO cohort (*n* = 46), where a familial history of cancer was available, 1/4 of patients with a germline P/LP variant (25%) had a known family history of breast and ovarian cancer at a young age. There were 5 (12%) other patients without a germline P/LP variant who had a family history of malignancy, 2 that were highly suggestive of an inheritable cancer predisposition.

## Discussion

**Figure 4.**
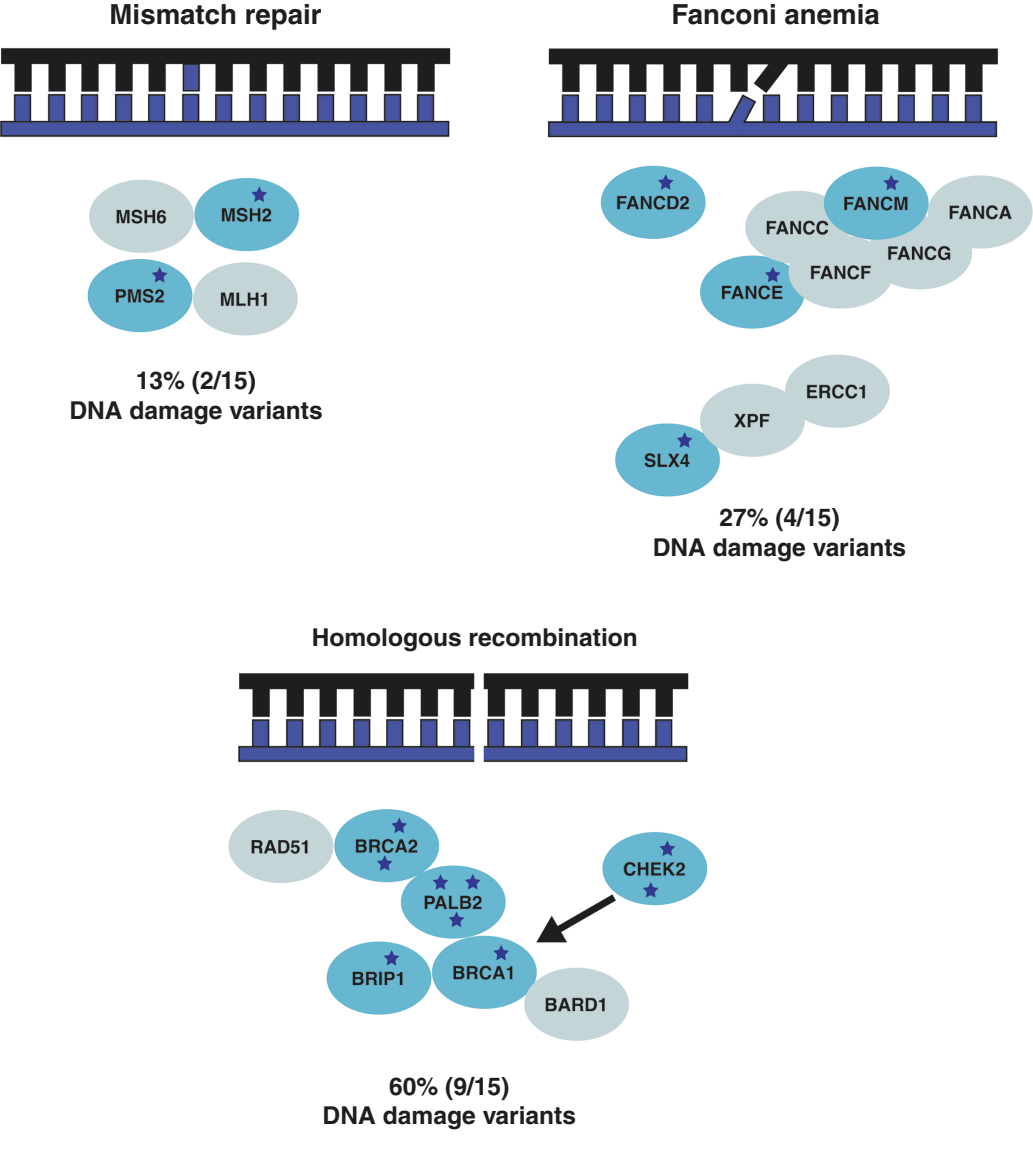
Germline variants in DNA damage response are enriched in diffuse midline glioma.. There were 15 variants detected in the DNA damage response pathway: 9 in homologous recombination, 4 in the Fanconi anemia pathway, 2 in mismatch repair. The 3 types of DNA damage response pathways altered in the diffuse midline gliomas cohort are demonstrated with an illustration of the type of DNA lesion that triggers the relevant pathway. The darker blue circles indicate genes with germline pathogenic/likely pathogenic (P/LP) variants detected in our cohort; and the dark blue stars are representative of a single patient with a germline P/LP germline variant. The lighter blue circles are some of the partner genes in the pathway that were unaffected in the cohort.

To the best of our knowledge, this is the largest international series of patients with DMG to assess the prevalence of germline P/LP variants in a tumor-agnostic list of CPGs. We found a prevalence of 7.5% of germline P/LP variants in CPGs, with enrichment in the HR and Fanconi anemia pathways (Figure 4). These deleterious germline variants were monoallelic except for one patient with an underlying pathogenic germline *FANCD2* variant with a second hit in the tumor due to chromosome 3 CN-LOH, leading to biallelic inactivation of *FANCD2* in the tumor. RNA sequencing suggested a complete loss of expression of HR genes *BRCA2* and *FANCE* in the tumor of a second patient. Thus definitive contribution of the germline variant to the somatic landscape was identified in 1% of patients. However, there appeared to be differences in the somatic landscape of patients with germline variants, with, for example, no patients in that group having somatic drivers in the PI3K/mTOR pathway. Preclinical modeling and a clinical case suggest that pathogenic germline mutations in HR pathway genes may lead to sensitization to PARP inhibition with potential therapeutic applications.

This international cohort has defined the prevalence of germline deleterious variants in DMG at 7.5%. Recent studies in childhood cancer patients found up to 16% prevalence of an underlying P/LP germline variant overall, with a 15% prevalence of germline P/LP variants across a heterogeneous CNS cohort.^7^ Our study, focused on DMG, yielded a lower germline prevalence of 7.5%. This difference may reflect the unique biological features of DMGs. Alternatively, while we included a comprehensive list of CPGs, it is possible that as yet undiscovered genes contribute to DMG risk.

Overall, while the prevalence found here is lower than in other childhood cancers, it is greater than the recommended 5% threshold indicating the need for clinical germline testing within a cancer type.^35^ Our results suggest that treating oncologists need to be aware of the potential association and to have a low threshold for germline testing in DMG patients with a family history of cancer.^35^ Consideration of screening for patients without a known family history could be supported by our data, as 75% of patients with a P/LP germline variant do not have a family history of malignancy. The limitations of screening based on family history alone have been described.^5,36^ Identification of a germline P/LP variant in known CPGs will require cascade testing and access to experienced clinical geneticists and genetic counselors.^37,38^

Furthermore, these results raise the question of whether deleterious germline variants contribute to tumorigenesis in DMG. We found definitive evidence of an impact of the germline on tumorigenesis in 1% of cases. Firstly, 2 of the 3 tumors with elevated signature 3 each had 2 pathogenic germline variants both in HR and Fanconi anemia genes (*BRCA2/FANCE*, *BRCA2/FANCD2*). The patient with germline *BRCA2* and *FANCE* pathogenic variants had RNA sequencing data that indicated the homozygous loss of *BRCA2* and *FANCE* in the tumor. When the mutational signature analysis was updated using COSMIC v3^25^, the tumor with an elevated signature 3 had 2 deleterious germline variants in the HR pathway *(BRCA2/FANCE*). This was the same patient who experienced a remarkable clinical response to PARP inhibition and who was 1 of the 2 patients who had a confirmed second hit in the tumor. Secondly, we found that there was a trend towards higher genome-wide LOH in tumors with germline P/LP variants in HR-related genes. While this effect was most pronounced in tumors with 2 germline P/LP variants, one in an HR-related gene and the second in a Fanconi anemia pathway gene, there was an effect seen on LOH across the cohort. Similarly, there was a significant difference in the somatic mutational landscape of those with germline P/LP variants with a remarkable absence of drivers in the PI3K/mTOR pathway in all cases. Taken together, these data suggest that germline variants contribute to oncogenesis in DMG in a subset of patients. Only 39 patients in our cohort had RNA sequencing data available, thus limiting a complete assessment of the somatic impact of germline findings for many patients. Further research is needed to understand more precisely how monoallelic germline HR variants impact oncogenesis. Additionally, the loss of *BRCA1* expression for one patient with elevated signature 3, without a molecular explanation, suggests that further study at a protein level could be helpful.

The germline findings in the HR pathway have potential therapeutic implications for DMG patients. The near complete radiologic response to olaparib and durvalamab, seen in one patient with multiply relapsed DMG and a germline pathogenic variant in *BRCA2* and *FANCE*, suggests the underlying germline landscape could have contributed to the treatment response. Immune checkpoint inhibitors have not been shown to be effective in DMG,^39,40^ therefore it is likely that the PARP inhibitor was an important driver of the clinical response, and that the response relates to HR deficiency in the tumor. Interestingly, a third patient had a loss of somatic *BRCA1* expression without identification of an underlying germline variant, suggesting other patients may have tumor landscapes susceptible to similar therapeutic strategies. Further preclinical and clinical studies could investigate the biological plausibility of oncogenesis due to the presence of a heterozygous pathogenic germline variant in the HR and MMR pathways.

Our study suggests some patients with DMG may benefit from PARP inhibition. The PARP inhibitor veliparib has previously been tested in DIPG in combination with radiation and temozolomide, without demonstrated treatment benefit.^41^ However patients in that trial were not selected based on somatic or germline biomarkers. Further work is needed to determine how many patients could potentially benefit from PARP inhibition, as the number of patients in published datasets in DIPG and high-grade gliomas with somatic homologous recombinant gene alterations is low.^2,3,15,16,42,43^ Individual variant data from the largest patient meta-analysis to date found that in DMG, 2/166 (1.2%) had somatic *BRCA2* and 5/166 (3.0%) had somatic *ATM* pathogenic variants, with 22/166 (13.3%) demonstrating somatic *ATRX* pathogenic variants.^3^ Somatic *BRCA2* pathogenic variants may be enriched in subtypes of DMG.^14^ Use of RNAseq and mutational signature data^44,45^ will enhance biomarker development and thus identify patients who may benefit from PARP inhibition. In addition, recent data demonstrates the differential potency of PARP trapping with agents such as veliparib (that have failed in DIPG clinical trials) being weak PARP trappers compared to talazoparib and olaparib.^46^ Indeed, a recent manuscript in pre-print supports a synergistic role for PARP inhibitors and radiotherapy across DMG models, using agents that are more efficient PARP trappers.^47^ They showed that defective homologous-recombination repair may be a direct effect of the *H3K27M* mutation itself, facilitating PARP inhibitor activity in unselected DMG.^47^ Continuous PARP inhibitor dosing and/or dosing based on pharmacodynamic endpoints will strengthen future trial designs to achieve maximum efficacy (via efficient PARP trapping) and to minimize toxicity.^48^

Our study found no cases with germline *TP53* P/LP variants among 252 DMG patients. This observation contrasts with other pediatric brain tumors such as glioblastomas, medulloblastomas, and choroid plexus carcinomas, where germline *TP53* variants are more frequently seen.^49–51^ Our findings are however consistent with previous studies reporting an absence of germline *TP53* variants in DMGs,^11,13,52^ noting isolated reports of germline *TP53* variants in DMG.^14,53^

Study limitations are the lack of availability of some clinical data points including family history, as well as somatic data for a small number of patients, to perform additional association analysis. Our study was restricted to the analysis of variants in coding and splice regions. Future work investigating the non-coding genome of this extensive DMG cohort could potentially lead to the identification of new causative variants. Non-coding variants are generally under-ascertained in clinical studies due to limited guidance on variant curation, functional impacts, and clinical reporting. Efforts to provide more guidance in this space will aid future analysis.^54^ We evaluated mutational signatures for those patients with available raw whole genome tumor sequencing data; however, a more comprehensive dataset could provide additional insight into the correlation between germline P/LP variants, particularly in the HR pathway, genome-wide LOH or other biomarkers that may predict PARP inhibitor response. Future work will focus on the expansion of the cohort to include more patients with multiomic data including RNA-seq and mutational signature.

Overall, the present study therefore has identified a prevalence of 7.5% of P/LP germline variants in the largest reported cohort of patients with DMG. The preclinical, clinical, and potential therapeutic applications are broad and will require appropriate focused studies to maximize knowledge for therapeutic and patient gain.

## Supplementary material

Supplementary material is available online at *Neuro-Oncology* (https://academic.oup.com/neuro-oncology).

noaf061_suppl_Supplementary_Table_S1_Figures_S1-S7

## Data Availability

Data will be made available upon reasonable request via our ZERO Data Access Committee or deposited in an online location once appropriate approvals are in place.
