# Supplementary material for: Germline analysis of an international cohort of pediatric diffuse midline glioma patients
Source: Neuro Oncol. 2025 Mar 12;27(7):1849–63. doi: 10.1093/neuonc/noaf061 (PMC12417819; doi:10.1093/neuonc/noaf061)
Supplement: noaf061_suppl_Supplementary_Table_S1_Figures_S1-S7 [file noaf061_suppl_supplementary_table_s1_figures_s1-s7.docx]

**Germline analysis of an international cohort of pediatric diffuse midline glioma patients**

**Mateos MK*, Ajuyah P* et al.**

**These authors contributed equally.*

[Supplemental methods 1](#_Toc186205099)

[Methylation analysis 1](#_Toc186205100)

[Germline gene of interest (GOI) 2](#_Toc186205101)

[Variant filtering 2](#_Toc186205102)

[Genome-wide loss of heterozygosity 2](#_Toc186205103)

[Generation of inducible lentiviral vectors for BRCA1 knockdown in cell lines 2](#_Toc186205104)

[Western blotting 3](#_Toc186205105)

[Supplementary Tables 4](#_Toc186205106)

[Supplementary Table 1. Germline genes of interest investigated in the present study. 4](#_Toc186205107)

[Supplementary Figures 8](#_Toc186205108)

[Supplementary Fig 1. H3K27M status and association with survival. 8](#_Toc186205109)

[Supplementary Fig 2. Deleterious germline variants mapped to known pathways. 9](#_Toc186205110)

[Supplementary Fig 3. Deleterious germline variants and association with survival. 10](#_Toc186205111)

[Supplementary Fig 4. Mutational signatures associated with homologous repair. 11](#_Toc186205112)

[Supplementary Fig 5. Methylation status of *BRCA1* and *BRCA2* 12](#_Toc186205113)

[Supplementary Fig 6. BRCA1 knockdown sensitizes DMG cells to PARP inhibition. 14](#_Toc186205114)

[Supplementary Fig 7. Germline workflow. 16](#_Toc186205115)

[References 18](#_Toc186205116)

# Supplemental methods

## Methylation analysis

The beta and M values for each probe was calculated and annotated against the hg19 genome build. Probes that were annotated to be within a CpG Island, promoter associated and annotated to *BRCA1* (11 probes) or *BRCA2* (2 probes) beta values were explored for association with the presence or absence of a germline mutation. Beta values closer to 1 corresponded with the probe being methylated and beta values closer to 0 unmethylated.

## Germline gene of interest (GOI)

Initially, a list of potentially relevant CPGs was identified by the most recent literature, including genes related to adult-onset CP. Selected genes were manually curated by a molecular scientist. Results were then validated by a second expert molecular scientists to assess validity. Discrepancies in assessment of potentially relevant genes were discussed in a multidisciplinary team (including, Cancer Geneticists, Molecular Scientists, Genetic Counsellors and Molecular Oncologists). Upon achieving consensus, the selected genes were included in the GOI list (Supplementary Table 1).

## Variant filtering

VCF files were first normalized by vt normalize (v0.5)(1) and annotated with VEP (v100) (2), followed by COSMIC (v95)(3), dbNSFP (v4.0a) (4, 5), ClinVar (2020-05-13)(6), dbSNP (2018-04-23)(7), ESP (ESP6500SI-V2)(8), GnomAD (v2.1.1) (9), MGRB (10) and CADD (v1.6) (11) using vcfanno (v0.3.2)(12). The annotated VCFs were converted into TSV files and variants that failed the following filters were excluded: gnomAD allele frequency <1%, those with VEP impact = LOW (generally where no change to the protein sequence is predicted); outside the genes of interest(13)(Supplementary Fig 4).

## Genome-wide loss of heterozygosity

In addition to the described methods, only one patient had both somatic *TP53* mutation and germline pathogenic variant in an HR-related gene, so the association between germline P/LP HR-related gene variants and LOH proportion was evaluated exclusively in the *TP53* wildtype cohort, using a single-tailed Wilcoxon rank sum test.

## Generation of inducible lentiviral vectors for BRCA1 knockdown in cell lines

After 48 h and 72 h, the viral supernatant was filtered and concentrated 10X using polyethylene glycol (PEG) precipitation (System Biosciences). The precipitated lentivirus was resuspended in DMEM with 25 mM HEPES and stored at -80°C. For the generation of stable cell lines, 300,000 SU-DIPGXIII cells were seeded in 6 well plates 24 h prior to lentiviral transduction. Cells were transduced with lentivirus in media containing 8µg/mL polybrene (Sigma) for 5 h, and were incubated with fresh media with no lentivirus overnight to recover. The following day, the cells were transduced for a second time for 5 h. 48 h after the second transduction, transduced cells were selected with 0.5µg/mL puromycin for 1 week. Cells were subsequently cultured in the presence of 0.25µg/mL puromycin to maintain selection.

## Western blotting

Cells were lysed in cell lysis buffer (Cell Signalling) supplemented with protease and phosphatase inhibitors (Roche) and protein concentration was determined using the Pierce BCA Protein Assay kit as per the manufacturer’s instructions. 20µg protein was then incubated for 5 minutes at 95°C in Laemmli loading buffer containing 10% DTT (Bio-Rad). Electrophoresis was conducted at 80-100V, and proteins were transferred to nitrocellulose membranes (BioRad) at 100V for 1 hour. Membranes were blocked in 5% skim milk in tris-buffered saline containing 0.05% Tween 20 (TBS-T) and then incubated with primary antibodies diluted in 5% blocking buffer overnight at 4°C. Antibodies used were: BRCA1 (1:1000, Cell Signalling #9010) and ß-Actin (1:1000, Cell Signalling). Membranes were washed 3x for 5 min each using TBS-T and incubated with anti-rabbit secondary antibody (1:2000, Cell Signalling) in blocking buffer for 1 hour at room temperature. Protein bands were visualised using chemiluminescence (Pierce) on a BioRad ChemiDoc imaging system.

# Supplementary Tables

## Supplementary Table 1. Germline genes of interest investigated in the present study.

Legend: Germline genes listed as “Green” were automatically included in the analysis, with subsequent variant filtration according to pathogenicity scoring and an in-house bioinformatic pipeline. (Table spans pp 2-5)

| **Gene name** | **Traffic light assignment** |
| --- | --- |
| ALK | Green |
| ANKRD26 | Green |
| APC | Green |
| ATM | Green |
| AXIN2 | Green |
| BAP1 | Green |
| BARD1 | Green |
| BLM | Green |
| BMPR1A | Green |
| BRCA1 | Green |
| BRCA2 | Green |
| BRIP1 | Green |
| CBL | Green |
| CDC73 | Green |
| CDK4 | Green |
| CDKN1B | Green |
| CDKN1C | Green |
| CDKN2A | Green |
| CEBPA | Green |
| CHEK2 | Green |
| CTR9 | Green |
| CXCR4 | Green |
| CYLD | Green |
| DDB2 | Green |
| DDX41 | Green |
| DICER1 | Green |
| DIS3L2 | Green |
| ELANE | Green |
| ELP1 | Green |
| EPCAM | Green |
| ERCC2 | Green |
| ERCC3 | Green |
| ERCC4 | Green |
| ERCC5 | Green |
| ETV6 | Green |
| EXT1 | Green |
| EXT2 | Green |
| FANCA | Green |
| FANCB | Green |
| FANCC | Green |
| FANCD2 | Green |
| FANCE | Green |
| FANCF | Green |
| FANCG / XRCC9 | Green |
| FANCI | Green |
| FANCL | Green |
| FH | Green |
| FLCN | Green |
| GATA2 | Green |
| GPC3 | Green |
| GPR161 | Green |
| GREM1 | Green |
| HRAS | Green |
| KIT | Green |
| KRAS | Green |
| LZTR1 | Green |
| MAX | Green |
| MEN1 | Green |
| MET | Green |
| MLH1 | Green |
| MPL | Green |
| MSH2 | Green |
| MSH6 | Green |
| MTAP | Green |
| MUTYH | Green |
| NBN | Green |
| NF1 | Green |
| NF2 | Green |
| NHP2 | Green |
| NRAS | Green |
| NSD1 | Green |
| NTHL1 | Green |
| PALB2 | Green |
| PAX5 | Green |
| PDGFRA | Green |
| PHOX2B | Green |
| PMS2 | Green |
| POLD1 | Green |
| POLE | Green |
| POT1 | Green |
| PRKAR1A | Green |
| PTCH1 | Green |
| PTEN | Green |
| PTPN11 | Green |
| RAD51 | Green |
| RAD51C | Green |
| RB1 | Green |
| RECQL3 | Green |
| RECQL4 | Green |
| REST | Green |
| RET | Green |
| RHBDF2 | Green |
| RUNX1 | Green |
| SBDS | Green |
| SDHA | Green |
| SDHAF2 | Green |
| SDHB | Green |
| SDHC | Green |
| SDHD | Green |
| SETBP1 | Green |
| SLX4 | Green |
| SMAD4 | Green |
| SMARCA4 | Green |
| SMARCB1 | Green |
| SMARCE1 | Green |
| SPRTN | Green |
| STK11 | Green |
| SUFU | Green |
| TERC | Green |
| TERT | Green |
| TMEM127 | Green |
| TP53 | Green |
| TRIM28 | Green |
| TSC1 | Green |
| TSC2 | Green |
| VHL | Green |
| WAS | Green |
| WRN | Green |
| WT1 | Green |
| XPA | Green |
| XPC | Green |
| ERCC1 | Green |
| FANCM | Green |
| KCNQ1OT1 | Green |
| PRF1 | Green |
| BUB1B | Green |
| CDH1 | Green |
| EGFR | Green |
| NOP10 | Green |
| RFWD3 | Green |
| RIT1 | Green |
| SRP72 | Green |
| STAT3 | Green |
| SHOC2 | Amber |
| RAF1 | Amber |
| AIP | Amber |
| FAS | Amber |
| FGFR2 | Amber |
| HOXB13 | Amber |
| MAP2K1 | Amber |
| MAP2K2 | Amber |
| RAD51D | Amber |
| BRAF | Amber |
| CREBBP | Amber |
| ENG | Amber |
| EP300 | Amber |
| IKZF1 | Amber |
| MITF | Amber |
| SOS1 | Amber |
| SPRED1 | Amber |

# Supplementary Figures

## Supplementary Fig 1. H3K27M status and association with survival.

Survival for patients with H3 K27 altered diffuse midline glioma (“Altered”) was compared to patients with H3 K27M wildtype diffuse intrinsic pontine glioma (“Wildtype”). There was a significant difference in overall survival (*P* = 0.022) between the two cohorts.

## Supplementary Fig 2. Deleterious germline variants mapped to known pathways.

Twenty-one germline pathogenic/likely pathogenic variants were detected in 19 patients. *MUTYH* is depicted here for completeness however was not considered a deleterious variant in our analysis. “HR”, homologous recombination; “MMR”, mismatch repair; “BER”, base excision repair; “MAPK-ERK”, “mitogen-activated protein kinase - extracellular-regulated kinase”; “RTK”, receptor tyrosine signalling. “H3K27 var”, H3K27 variant; “Mutant” refers to patients with a known somatic H3K27M mutation who do not have further details regarding the type of histone mutation; “Altered” refers to H3K27 altered without H3K27M mutation; “WT”, wildtype.

## Supplementary Fig 3. Deleterious germline variants and association with survival.

Survival for patients with an underlying pathogenic germline variant (“Yes”) was compared to those without an underlying pathogenic germline variant (“No”). There was no difference between overall (Panel A) or progression-free survival (Panel B) between the two cohorts.


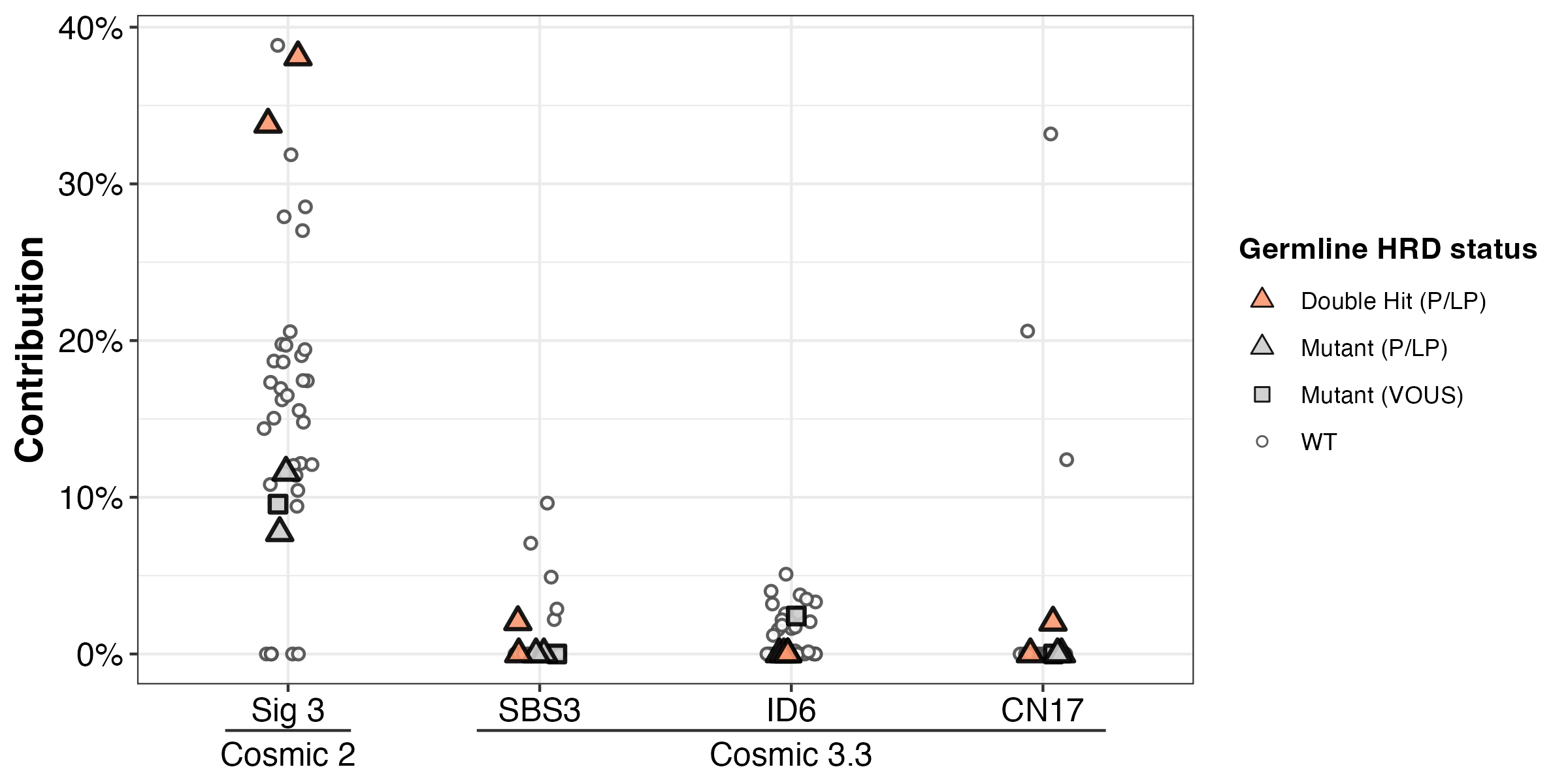


## Supplementary Fig 4. Mutational signatures associated with homologous repair.

We assessed how each patient with a germline P/LP variant scored on relevant mutational signatures (as a % contribution). The contribution is shown for COSMIC v2 and COSMIC v3.3. The three COSMIC v3.3 signatures scores assessed were single base substitution (SBS), indel (ID), and copy number (CN) signatures, respectively. Patients with an underlying germline P/LP variant in an HR-related gene (termed homologous recombinant deficiency (HRD) in this figure) are depicted as triangles, and colored orange for the two patients harbouring multiple P/LP germline HRD mutations (“double hit”). “VOUS”, variant of unknown significance.

## Supplementary Fig 5. Methylation status of *BRCA1* and *BRCA2*

Heatmap representation of the significant differentially methylated CpG islands in *BRCA1* and *BRCA2* are shown with the corresponding beta value for each diffuse midline glioma patient with methylation data. Methylation status of the *BRCA1* and *BRCA2* body and promoter was examined for patients with and without germline homologous recombination deficiency (HRD), showing no difference between the groups. The beta value is a range between 0-1 with 0 (blue) being unmethylated, 1 (red) methylated and 0.5 (white). CpG islands therefore identified as unmethylated are blue and methylated are red. The genomic coordinates are genome build hg19. “VUS”, variant of unknown significance.

## Supplementary Fig 6. BRCA1 knockdown sensitizes DMG cells to PARP inhibition.

(A) Western blots show that *BRCA1* knockdown (KD) was achieved using two separate BRCA1 shRNA doxycycline-induced lentiviral constructs (*BRCA1* shRNA#1, *BRCA1* shRNA #2). (B, C) *BRCA1* KD led to increased sensitivity to niraparib (PARP inhibitor) monotherapy, which was not seen in *BRCA1* wildtype cells (** indicates significant using two-way ANOVA with Sidak’s multiple comparison test; *BRCA* shRNA#1 *P*=0.005*; BRCA* shRNA #2 *P*=0.067). Dox, doxycycline; Nirap., niraparib.

## Supplementary Fig 7. Germline workflow.

The workflow for the present study and incorporation of the international cohorts is depicted. Germline single nucleotide variant (SNV) and short insertion-deletions (indel) analysis was performed across the cohort. Germline copy number variant (CNV) analysis was also performed on Australian ZERO patients. *Additional data processed within the ZERO cohort included RNA sequencing and methylation, enabling assessment of mutational signatures, methylation of gene promoters (e.g *BRCA1/BRCA2*) and genome-wide loss of heterozygosity (LOH). WGS, whole genome sequencing; WES, whole exome sequencing.

# References

1. Tan A, Abecasis GR, Kang HM. Unified representation of genetic variants. Bioinformatics. 2015;31(13):2202-4.

2. McLaren W, Gil L, Hunt SE, Riat HS, Ritchie GRS, Thormann A, et al. The Ensembl Variant Effect Predictor. Genome Biology. 2016;17(1):122.

3. Tate JG, Bamford S, Jubb HC, Sondka Z, Beare DM, Bindal N, et al. COSMIC: the Catalogue Of Somatic Mutations In Cancer. Nucleic Acids Research. 2018;47(D1):D941-D7.

4. Liu X, Jian X, Boerwinkle E. dbNSFP: A lightweight database of human nonsynonymous SNPs and their functional predictions. Human Mutation. 2011;32(8):894-9.

5. Liu X, Li C, Mou C, Dong Y, Tu Y. dbNSFP v4: a comprehensive database of transcript-specific functional predictions and annotations for human nonsynonymous and splice-site SNVs. Genome Medicine. 2020;12(1):103.

6. Landrum MJ, Lee JM, Benson M, Brown GR, Chao C, Chitipiralla S, et al. ClinVar: improving access to variant interpretations and supporting evidence. Nucleic Acids Research. 2017;46(D1):D1062-D7.

7. Sherry ST, Ward M-H, Kholodov M, Baker J, Phan L, Smigielski EM, et al. dbSNP: the NCBI database of genetic variation. Nucleic Acids Research. 2001;29(1):308-11.

8. Exome Variant Server, NHLBI GO Exome Sequencing Project (ESP) Seattle, WA, USA [Available from: <http://evs.gs.washington.edu/EVS/> Dec 2021 accessed].

9. Karczewski KJ, Francioli LC, Tiao G, Cummings BB, Alföldi J, Wang Q, et al. The mutational constraint spectrum quantified from variation in 141,456 humans. Nature. 2020;581(7809):434-43.

10. Medical Genome Reference Bank – Data Access [Available from: <https://sgc.garvan.org.au/initiatives/mgrb> accessed Dec 2021].

11. Rentzsch P, Schubach M, Shendure J, Kircher M. CADD-Splice—improving genome-wide variant effect prediction using deep learning-derived splice scores. Genome Medicine. 2021;13(1):31.

12. Pedersen BS, Layer RM, Quinlan AR. Vcfanno: fast, flexible annotation of genetic variants. Genome Biology. 2016;17(1):118.

13. Cunningham F, Allen JE, Allen J, Alvarez-Jarreta J, Amode M R, Armean Irina M, et al. Ensembl 2022. Nucleic Acids Research. 2021;50(D1):D988-D95.

End of supplementary.
